# Selective vulnerability to neurodegenerative disease: the curious case of Prion Protein

**DOI:** 10.1242/dmm.012146

**Published:** 2014-01

**Authors:** Walker S. Jackson

**Affiliations:** German Center for Neurodegenerative Diseases (DZNE), Sigmund-Freud-Str. 25, 53127-Bonn, Germany.

**Keywords:** Huntington’s disease, Neurodegeneration, Spinocerebellar ataxia, Knock-in mice, Neuropathology, Prion diseases

## Abstract

The mechanisms underlying the selective targeting of specific brain regions by different neurodegenerative diseases is one of the most intriguing mysteries in medicine. For example, it is known that Alzheimer’s disease primarily affects parts of the brain that play a role in memory, whereas Parkinson’s disease predominantly affects parts of the brain that are involved in body movement. However, the reasons that other brain regions remain unaffected in these diseases are unknown. A better understanding of the phenomenon of selective vulnerability is required for the development of targeted therapeutic approaches that specifically protect affected neurons, thereby altering the disease course and preventing its progression. Prion diseases are a fascinating group of neurodegenerative diseases because they exhibit a wide phenotypic spectrum caused by different sequence perturbations in a single protein. The possible ways that mutations affecting this protein can cause several distinct neurodegenerative diseases are explored in this Review to highlight the complexity underlying selective vulnerability. The premise of this article is that selective vulnerability is determined by the interaction of specific protein conformers and region-specific microenvironments harboring unique combinations of subcellular components such as metals, chaperones and protein translation machinery. Given the abundance of potential contributory factors in the neurodegenerative process, a better understanding of how these factors interact will provide invaluable insight into disease mechanisms to guide therapeutic discovery.

## Introduction

Neurodegenerative diseases affect tens of millions of people worldwide every year. Trying to limit this devastating assault on human health is one of the most pressing challenges in current medicine. Neurodegenerative diseases, which are generally lethal, are typified by the physical decay and eventually loss of neurons. Like cancer, neurodegenerative disorders are a phenotypically heterogeneous group of diseases, with each having unique characteristics; however, several key features are shared. All neurodegenerative diseases are thought to be caused by the misfolding of specific proteins and the eventual clumping of misfolded proteins into aggregates, as the disease progresses. Although the aggregates found in histological sections of brains affected by different neurodegenerative diseases have different shapes and tinctorial properties, they seem to develop from a common pathway, as demonstrated by their universal reactivity with a pair of antibodies generated against an Alzheimer’s disease (AD)-related peptide (Kayed et al., 2003). The antibodies react with either small oligomeric forms or higher-order aggregates from several neurodegenerative disease-related proteins, including prion diseases (PrDs) (Aidt et al., 2013), indicating that the misfolded proteins share common conformational transition states (Glabe, 2006; Kayed et al., 2003). Inherited mutations in the genes encoding many of these proteins are causally linked to familial forms of neurodegeneration, further highlighting the importance of the malformed proteins for disease development. Neurodegenerative diseases generally present clinical signs at mid-late life, which is intriguing in the case of familial neurodegenerative diseases, in which the disease-causing mutant protein is present throughout life. Pathological changes commonly observed in neurodegenerative diseases include reactive astrocytosis and microgliosis (Aguzzi et al., 2013; Sofroniew and Vinters, 2010). With so many similarities between the different neurodegenerative diseases, the knowledge gained from studying one can often be applied to others. Nonetheless, there are many differences between the diseases, and understanding the mechanisms involved will be crucial for designing therapies. However, there are many pieces to the puzzle, making this an extremely challenging problem.

One reason this is an enormous challenge is because the brain is extraordinarily complex. It is built of highly interconnected networks of numerous neural nuclei. These nuclei consist of many types of neurons and, in most neurodegenerative diseases, only a subset of neurons in specific nuclei are initially targeted. For example, cholinergic neurons of the cerebral cortex as well as hippocampal neurons are targeted in AD (Francis et al., 1999), whereas dopaminergic neurons of the substantia nigra are targeted in Parkinson’s disease (PD) (Sulzer and Surmeier, 2013). Spinocerebellar ataxia-1 (Sca1) and Huntington’s disease (HD), two members of a broad class of diseases linked to long CAG (encoding glutamine)-repeat mutations, both affect GABAergic neurons [utilizing γ-aminobutyric acid (GABA)]. The cell populations affected, however, differ: Sca1 targets giant Purkinje cells of the cerebellum (Zoghbi, 2000), whereas HD targets medium-sized spiny neurons of the striatum (Graveland et al., 1985). Interestingly, the genes causing Sca1 and HD are expressed in both regions, implying that a single mutation, the long CAG repeat, can cause a number of diseases depending on which gene carries it. The reasons that both regions are not affected in both diseases are poorly understood.

**Fig. 1:**
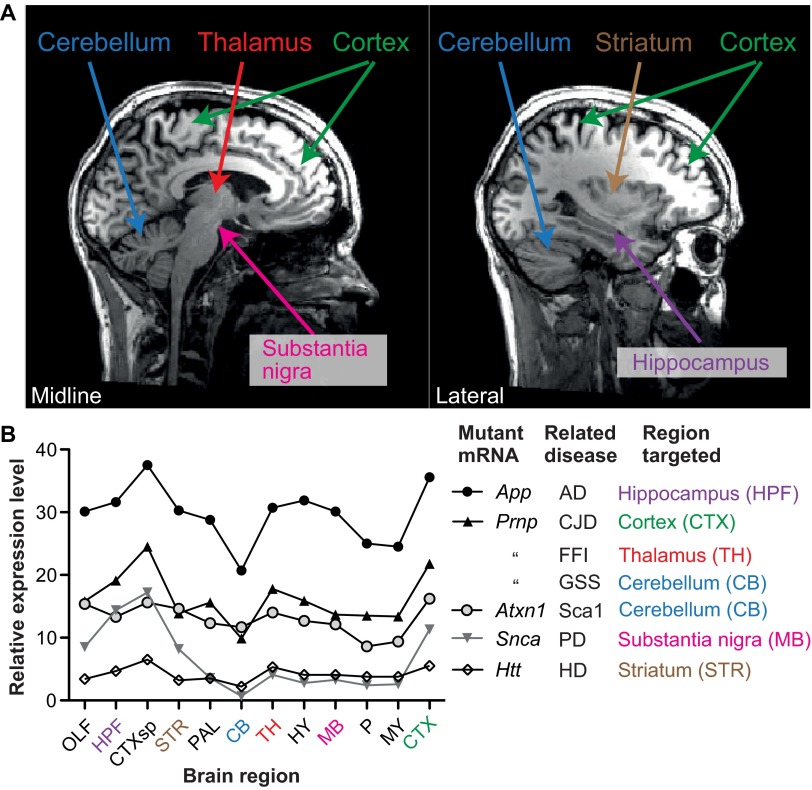
**Different brain regions affected by different familial PrDs.** (A) A powerful technique employed by neurologists to diagnose disease is to non-invasively look at a patient’s brain using magnetic resonance imaging (MRI). Two images in the sagittal plane from a healthy individual facing right are used as maps to indicate the regions affected in various neurodegenerative diseases and to highlight that the regions are widely distributed. The image on the left is at the mid-line, the other image is parallel but towards one side. (B) A line chart showing the spatial distribution of mRNAs that cause specific neurodegenerative diseases when mutated. These data were measured by *in situ* hybridization of the adult mouse brain. Expression levels are not appreciably higher in the areas that are targeted. Data were acquired from the Allen Brain Atlas website (www.brain-map.org). Brain region abbreviations: OLF, olfactory bulb; HPF, hippocampus; CTXsp, subregion of the cortex (bottom of the brain); STR, striatum; PAL, pallidum; CB, cerebellum; TH, thalamus; HY, hypothalamus; MB, midbrain; P, pons; MY, medulla; CTX, cortex (main region on top of the brain).

Selective vulnerability is also a hallmark feature of the PrDs. In this group of disorders, GABAergic neurons are also affected, yet the diseases have some important features that distinguish them from the CAG-repeat diseases. Aside from the frightening names of some, such as chronic wasting disease, ‘mad cow’ disease and fatal familial insomnia (FFI), PrDs are infamous for the cases where disease has spread between individuals, sometimes across species (Prusiner, 1998). The causative agent is believed to be a prion: an infectious agent formed of abnormal, misfolded protein. Practices that facilitate the spread of PrDs between individuals include medical procedures involving contaminated tools or tissues, consumption of contaminated food and human cannibalism (Prusiner, 1998). A less well-known, but arguably more interesting, characteristic is that PrDs can have quite disparate clinical features (Kovács et al., 2002). Familial PrDs, which are all caused by dominant mutations in the protein-coding sequence of the same gene, *PRNP*, encoding the prion protein (PrP), are emblematic of this clinical pleiotropy. These diseases, caused by >30 different mutations, are classified into three types based on a combination of clinical and neuropathological changes (Kovács et al., 2002; Rossetti et al., 2011). Creutzfeldt-Jakob disease (CJD) generally presents as a cognitive disease characterized by severe neuronal loss, spongiform degeneration (spongiosis) and amorphous PrP aggregates, and primarily targets the cortex (Fig. 1) (Gambetti et al., 2003; Gambetti et al., 1995). Gerstmann-Straussler-Scheinker syndrome (GSS) is a motor system disease in which the cerebellum (Fig. 1) is affected by amyloid PrP aggregates (stainable with special dyes such as thioflavin T or Congo red); however, these brains generally show little neuronal loss or spongiosis (Ghetti et al., 1994; Piccardo et al., 1998). Finally, FFI, which is characterized by disruptions in sleep homeostasis and autonomic nervous system functions, primarily targets the thalamus (Fig. 1), with severe neuronal loss and reactive gliosis but relatively little PrP aggregation or spongiosis (Gambetti et al., 2003; Gambetti et al., 1995). Although individual mutations cause some additional, unique features that can distinguish their associated phenotypes from others assigned to the same disease type and, furthermore, the different disease types have some overlap (for example, CJD and GSS can both affect the cortex and cerebellum, but to a different extent), mutations in *PRNP* cause diseases that are consistently assigned to one of these three disease types (Kovács et al., 2002). Thus, familial PrDs contrast with CAG-repeat diseases because a single gene can cause multiple diseases depending on which mutation it carries. It is important to note that once a disease has progressed for many months or years, it spreads into new areas (Braak and Braak, 1991) and the selectivity technically diminishes. However, the diseases start in a specific area (Braak and Braak, 1991; Graveland et al., 1985; Hyman et al., 1984) and understanding what factors determine this selectivity is not only scientifically fascinating, but also medically important, because early interventions are likely to be the most efficacious.

One potential explanation for selective vulnerability is that the gene that triggers protein misfolding is expressed at higher levels in areas that are affected the most. However, this hypothesis is unlikely to be vindicated because many neurodegenerative disease-related genes have similar levels of expression in both affected and unaffected areas (Fig. 1). Moreover, this cannot be the case for PrDs because, as mentioned, mutations in a single gene give rise to all the disease variants. Indeed, this makes the study of familial PrD pathology particularly important because they are all caused by mutations in *PRNP*, and comparisons between different diseases are not confounded by expression pattern differences. Here, I discuss several factors that might influence the selective targeting of brain regions in different diseases, using familial PrDs as a model.

## The role of *PRNP* mutations in selective vulnerability

PrP is initially translated as a 254 amino acid polypeptide that is translocated into the endoplasmic reticulum and passages through the Golgi apparatus during synthesis (Taraboulos et al., 1992). Eventually, it is retained with a glycosyl-phosphatidylinositol anchor in an extracellular lipid raft environment of the cell membrane (Stahl et al., 1987). The mature form is a 208 amino acid globular glycoprotein with an unstructured N-terminus (Riek et al., 1996; Riek et al., 1997), two glycan chains (Haraguchi et al., 1989) and a disulfide bridge (Turk et al., 1988). [See Riesner (Riesner, 2003) for an intelligently designed schematic.] Many roles have been proposed for PrP, including the maintenance of neural stem cells (Steele et al., 2006) and myelin sheath (Bremer et al., 2010), metal homeostasis (Brown et al., 1997; Kralovicova et al., 2009; Pushie et al., 2011), modulation of NMDA (N-methyl-D-aspartate) receptors (Khosravani et al., 2008), and protection from ischemia-induced degeneration (Weise et al., 2006). It was recently proposed to be a receptor for a toxic protein fragment in AD (Laurén et al., 2009; Resenberger et al., 2011), but this hypothesis remains controversial (Calella et al., 2010; Larson et al., 2012). All of these important aspects of prion biology could potentially drive the selective vulnerability of familial PrDs; however, at present there is no overriding theory.

A logical strategy to understand how *PRNP* mutations target specific brain regions is to search for underlying patterns that link individual mutations with features of the diseases they cause (Capellari et al., 2011). One possibility is that mutations destabilize PrP to various extents, and the more severely PrP is misfolded, the more rapidly the disease progresses and the more widely distributed the neuropathological lesions, assuming that there would be a higher proportion of cells that cannot tolerate the misfolded protein. However, there is strong evidence to refute this hypothesis (Capellari et al., 2011). A mutant that strongly destabilizes PrP (F198S, Table 1) results in a late-onset, rather slowly developing PrD, whereas mutants causing moderate (D178N) or no (E200K) destabilization cause earlier and faster-progressing diseases (Apetri et al., 2004; Capellari et al., 2011; Kovács et al., 2002). Mutations also occur in the unstructured N-terminal region, including substitutions of prolines for leucines and increases or decreases in the size of an octapeptide repeat (Piccardo et al., 1998). Interestingly, the clinical phenotypes associated with the N-terminal mutations include GSS, CJD and even an HD-like disease. Because these mutations should have no influence on the stability or three-dimensional structure, and yet they target different brain regions, factors other than destabilizing changes to PrP must be able to direct selective vulnerability.

**Table 1:**
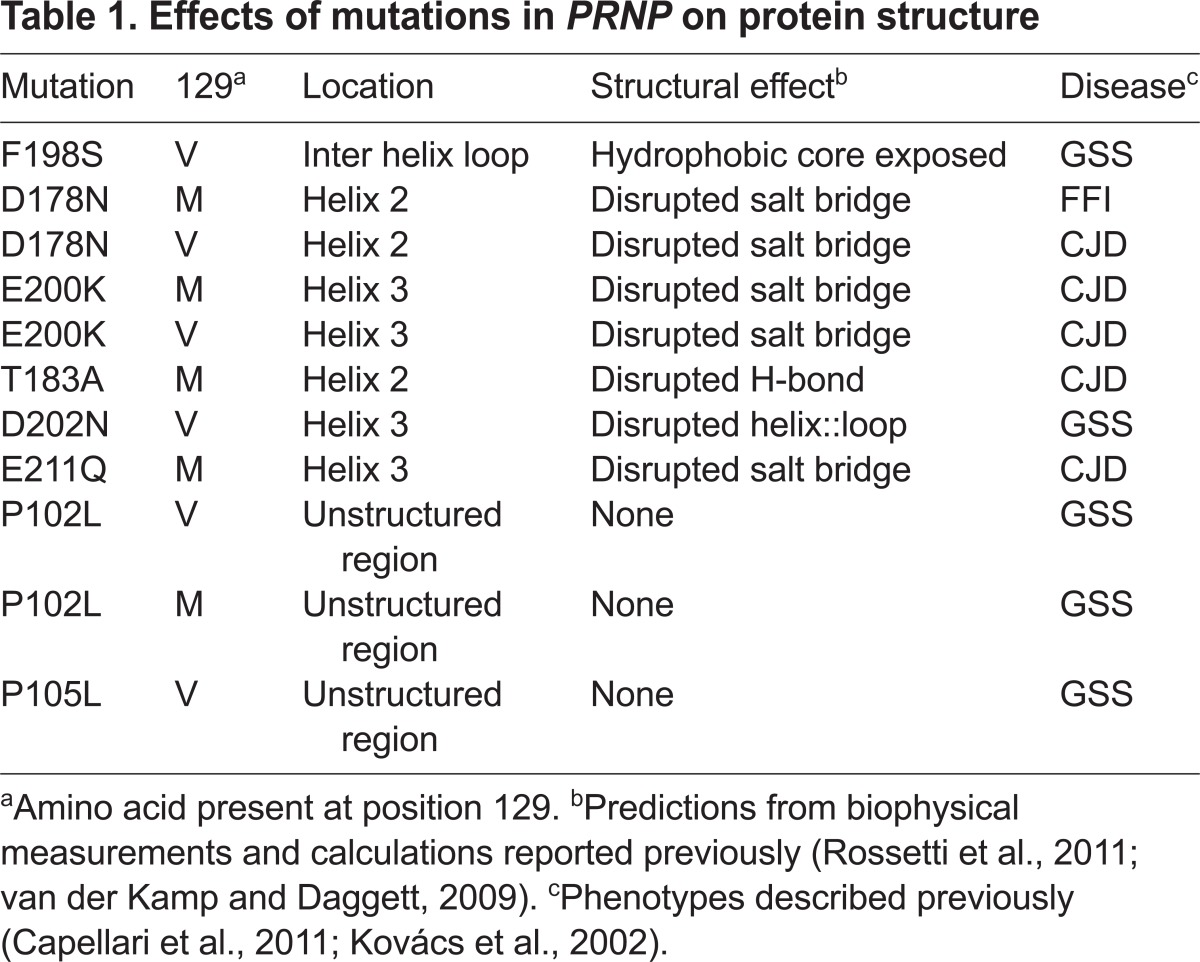
Effects of mutations in *PRNP* on protein structure

Changes in the overall protein charge could also drive selective vulnerability, by favoring aggregation pathways associated with a specific PrD subtype. Indeed, this would explain why over half of the mutations identified for PrDs involve changes in charge (Rossetti et al., 2011; Shen and Ji, 2011). However, mutants resulting in the same charge change can cause different diseases. For example, D178N causes FFI, D202N causes GSS and E211Q causes CJD, yet all of these mutations involve the substitution of a negatively charged amino acid with a polar uncharged amino acid (Capellari et al., 2011; Kovács et al., 2002). Moreover, mutations without charge changes (e.g. T183A and F198S) can also cause different diseases (CJD and GSS, respectively).

Interestingly, a very common polymorphism affecting amino acid position 129 in the PrP polypeptide chain, which does not itself cause disease, can modify the effects of disease-causing mutations. Presence of the D178N mutation together with a methionine at amino acid 129 (129M) causes FFI and targets the thalamus, but, when the same mutation is in cis with valine at amino acid 129 (129V), the thalamus is relatively unscathed and instead the cortex is targeted, resulting in CJD. In contrast, compared with the 129M variant, 129V in cis with the E200K mutation steers pathology towards the thalamus, but still causes CJD. The only trend between sequence changes and disease phenotype is that most cases of GSS include 129V on the mutant allele, but the presence of this polymorphism in some familial CJD cases diminishes the correlation. The fact that this polymorphism at amino acid 129 without a charge change partially determines which brain regions are targeted indicates that factors other than charge differences are driving selective vulnerability. Therefore, no clear pattern exists, such as extent of destabilization of the native structure, position in the linear sequence or charge change, which explains how each mutation causes a specific disease. It seems that different mutations lead PrP to form distinct misfolded conformers or aggregates, and each of these unique structures somehow targets specific regions.

Interest in studying the familial PrDs has led to the development of several mouse models. Many carry randomly integrated transgenes expressing the mutant protein, an approach that generally leads to expression levels that are much higher than endogenous levels (Dossena et al., 2008; Friedman-Levi et al., 2011; Hsiao et al., 1990; Yang et al., 2009). However, overexpression of wild-type PrP is toxic to mice (Chiesa et al., 2008; Watts et al., 2012; Westaway et al., 1994), making it difficult to distinguish the effects of the mutation from any confounding effects associated with overexpression in mouse models designed this way. Another problem is that the spatial expression patterns are highly dependent on the genomic integration site, vary from line to line and do not accurately mimic the endogenous expression pattern (Borchelt et al., 1996; Faas et al., 2010; Karapetyan et al., 2009).

**Fig. 2:**
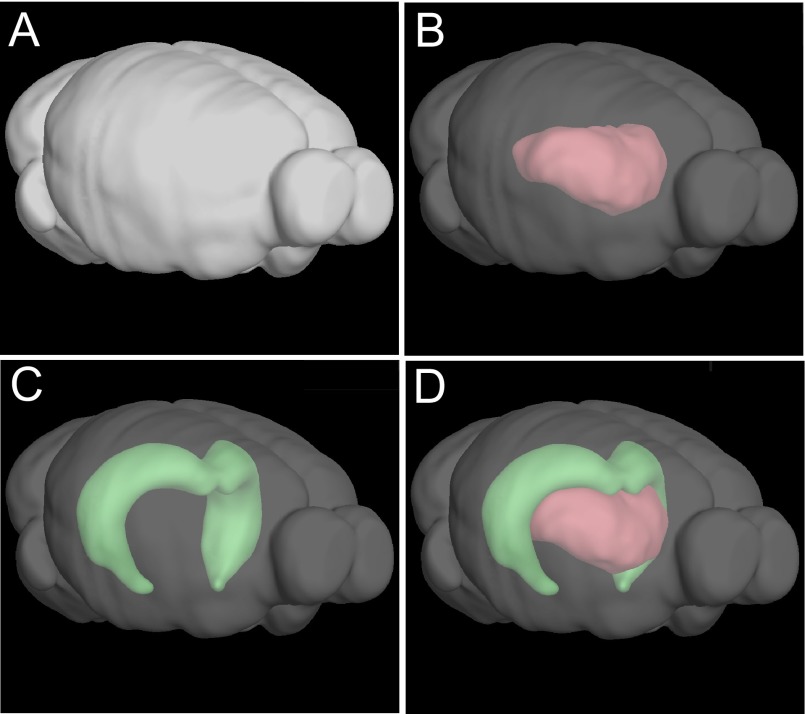
**The thalamus and hippocampus are intertwined in the mouse brain.** (A) A computer rendering of the mouse brain oriented so that, in an intact mouse, it would be facing slightly to the right of the observer. (B) The same model as in A but with the external regions made translucent so that the space occupied by the thalamus can be observed. (C) The same model as in B but with the hippocampus highlighted instead. (D) The hippocampus and thalamus shown together. These images were created on the Allen Brain Atlas website (www.brain-map.org).

In recent years we have been developing an allelic series of knock-in mice to model six of the most interesting familial PrDs. The knock-in approach introduces mutations to the endogenous gene at the site that it naturally exists in the genome, thereby facilitating endogenous levels of expression. Two lines, D178N-129M and E200K-129M, which model FFI or CJD, respectively, show selective vulnerability, with the former targeting the thalamus and the latter targeting the hippocampus (Jackson et al., 2009; Jackson et al., 2013). These two structures are in very close proximity in the brain: the hippocampus, which resembles a partial shell, virtually cups the thalamus, which is shaped roughly like a disc (Fig. 2). However, they have few neuroanatomical connections (Somogyi and Klausberger, 2005). How these two mutations, located just 60 base pairs away in the context of the 3-billion base pairs of mouse genome, encode such specific targeting in the brain is currently under intensive investigation in our group.

Tau is a microtubule protein that has been implicated in the pathogenesis of neurodegenerative diseases, including PrDs. Like PrP, mutations affecting Tau are associated with clinical pleiotropy (Wolfe, 2009). An allelic series of *Tau* knock-in mice carrying mutations linked to familial human diseases with different neuropathological changes could also be an invaluable tool for neurodegenerative disease research.

## Brain factors determining selective vulnerability

Because mutant proteins cause different problems in different brain regions, brain-region-specific factors must play a role in determining selective vulnerability. One possible factor is that intrinsic firing properties or metabolic activity of specific cell types might make specific regions more vulnerable to degeneration. For example, GABA neurons, mentioned earlier as being affected in CAG-repeat diseases and PrDs, are inherently highly active cells. The constant firing is energetically expensive because of the frequent need to reestablish ion gradients. The resulting high metabolic rate could prime these cells for damage due to the high production of free radicals. The overworked cellular components would become prone to damage, requiring more degradation and re-synthesis of cellular components and further increased metabolic demand, setting a vicious cycle into motion. As the brain ages, it would likely struggle to cope with the heavy burden of degrading damaged cellular components and making repairs, particularly in the presence of a misfolded protein. A similar argument was made for the subset of dopaminergic neurons that have an intrinsically high firing rate and are selectively targeted in PD (Chan et al., 2007). Data from mouse models, however, suggest that there is more to the story. Knock-in mutations in mice modeling HD, Sca1 and FFI primarily affect the striatum, cerebellum and thalamus, respectively (Jackson et al., 2009; Lin et al., 2001; Watase et al., 2002), just as in humans, suggesting that the mouse lines model the corresponding diseases relatively well. However, the mouse thalamus has a far lower proportion of GABAergic neurons compared with the human thalamus (Arcelli et al., 1997). This leads to an alternative hypothesis, at least for FFI, that their location in the brain, rather than simply their neurochemical or intrinsic firing properties, can influence a neuron’s vulnerability. In addition, non-neuronal cells (e.g. glial and vascular cells) are also involved in the disease process and could cause a mixture of positive and negative effects, both of which would influence selective vulnerability. There are many unanswered questions in this regard, highlighting the need for further research in this area.

Metal ions might also play an important role in making certain brain regions more vulnerable to neurodegenerative diseases. Maintenance of the appropriate stoichiometry of metals and their biomolecular partners is critical because metals are required for numerous synthesis, degradation and defensive processes, and can be toxic in free forms. Like the non-uniform distribution of neuron types, metals also have variable concentrations across brain regions and, interestingly, the levels of three common metal ions, Cu^2+^, Fe^2+^ and Zn^2+^, seem to be regulated by PrP in normal brains (Brown et al., 1997; Kralovicova et al., 2009; Pushie et al., 2011; Watt et al., 2012). Moreover, Cu^2+^ and Mn^2+^ levels change in PrDs (Hesketh et al., 2008; Thackray et al., 2002). In addition to PrP-mediated modification of metal concentrations, the reciprocal is also true, i.e. metals can modify the activities of PrP. For example, the inhibitory effect of PrP on NMDA-receptor activity occurs via a Cu^2+^-dependent mechanism (You et al., 2012). Interestingly, changes in metal levels change PrP levels (Kralovicova et al., 2009) as well as redirecting its trafficking through the secretory pathway (Brown and Harris, 2003). In support of a role for metal ions in modulating PrP function in the context of disease, excess Cu^2+^ aggravates disease in a mouse model of familial CJD (Canello et al., 2012). Furthermore, Zn^2+^ interacts with PrP to varying degrees depending on the mutation present, with weaker interactions being associated with mutations conferring charge changes (Spevacek et al., 2013). Therefore, differences in metal concentrations are likely to modify regional sensitivity to different PrP variants, but understanding how and to what extent this happens awaits further research.

**Fig. 3:**
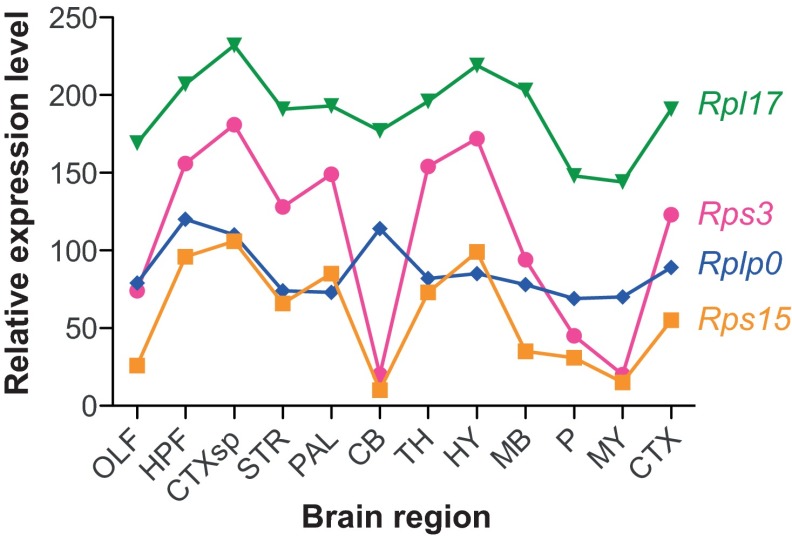
**A surprising distribution of ribosomal protein mRNAs in the brain.** Small and large ribosomal subunit proteins are encoded by Rps and Rpl mRNAs, respectively. A small subunit binds to mRNAs first and recruits the large subunit for translation. A variable expression pattern of components of both subunits in the mouse brain suggests the existence of extra layers of gene regulation that were previously unrecognized. Data were acquired from the Allen Brain Atlas website (www.brain-map.org). The data were systematically normalized and therefore comparable to the mRNA distributions in Fig. 1. Abbreviations: OLF, olfactory bulb; HPF, hippocampus; CTXsp, subregion of the cortex (bottom of the brain); STR, striatum; PAL, pallidum; CB, cerebellum; TH, thalamus; HY, hypothalamus; MB, midbrain; P, pons; MY, medulla; CTX, cortex (main region on top of the brain).

Another factor potentially controlling selective vulnerability is that ubiquitous molecular machines that reduce or restrict toxicity, for example components of the protein-folding and quality-control systems, might vary between brain regions. Indeed, different strains of mouse prions causing different morphologies of aggregates and targeting different brain regions activate different combinations of chaperones (Asuni et al., 2013). Moreover, in the context of cell culture experiments, where a small number of cell lines is typically employed, PrP mutants associated with different diseases can have similar trafficking abnormalities, suggesting that trafficking defects cannot contribute to selective vulnerability (Ashok and Hegde, 2009; Capellari et al., 2000a; Capellari et al., 2000b; Ivanova et al., 2001; Petersen et al., 1996; Zou et al., 2011). However, the massive diversity of cell types in the brain provides an exponentially greater number of microenvironments for mutant PrPs to interact with. Thus, if the protein quality-control system has distinct combinations of components expressed in specific cell types, then some cells might be less capable of dealing with a specific misfolded conformer than others, thereby being more vulnerable to disease. Inconsistent with this hypothesis, mRNAs encoding protein chaperones are expressed at approximately equal levels across the adult mouse brain (Tebbenkamp and Borchelt, 2010), suggesting that there is little cell-type specificity for this critical system, and all cells should be equally capable of detoxifying a misfolded protein. However, the relative expression levels of ribosomal protein mRNAs vary across the body of the developing mouse, suggesting that levels of translation could vary (Kondrashov et al., 2011). Examination of ribosomal protein mRNAs in the adult mouse brain using data from the Allen Brain Atlas (www.brain-map.org) corroborates this, revealing a startling variability in expression between regions (Fig. 3). Some ribosomal protein mRNAs are approximately uniformly distributed across the brain (Fig. 3, Rpl17 and Rplpo), whereas others are highly variable (Fig. 3, Rps3 and RPS15). Moreover, of the 62 ribosomal genes examined, at least seven are apparently not expressed in the adult mouse brain (Rpl12, Rpl27, Rpl37, Rpl41, Rps13, Rps17, Rps20). The non-stoichiometric distribution of ribosomal protein mRNAs suggests that there is a large diversity of ribosomal configurations, distributed across specific cell types, which could impact the regulation of translation. Therefore, the chaperone repertoire of specific cell types in healthy brains is likely to be much more specialized than is reflected by Tebbenkamp and Borchelt’s mRNA localization study, and the same is probably true for components of other processes, including degradation and synthesis. Because the stressful conditions of acquired PrD cause protein translation to be sharply altered (Moreno et al., 2012), the molecular composition of each cell is likely to be drastically reshaped during disease.

A better understanding of how the multitude of various components of cells (e.g. protein quality-control machinery) are distributed across the brain, in the presence and absence of disease, is required to fully understand how these factors make certain regions most vulnerable.

## Hitting a moving target

**Fig. 4:**
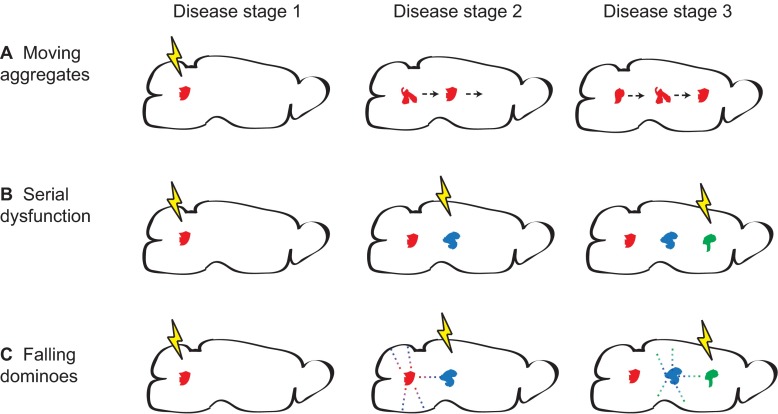
**Models of protein aggregate spread.** Three models of how the localization of aggregates in the brain might change as neurodegenerative disease progresses. (A) Moving aggregates. Aggregates generated in one region physically move to another, covering a large distance. (B) Serial dysfunction. Due to network dysfunction, conditions inducing *de novo* synthesis of protein aggregates move across the brain. (C) Falling dominoes. Similar to serial dysfunction, where conditions permissive to aggregate formation move, but with the distinction that misfolded protein aggregates seed the formation of new aggregates in close proximity, creating a chain reaction. The lightning bolts represent induction of conditions in different brain regions that accommodate the appearance of protein aggregates. Conditions might include aberrant network activity or impairment of protein quality-control machinery. The arrows in A show that an aggregate formed in the left end of the brain moves towards the right end. The dots in C represent small oligomeric misfolded protein seeds, which spread and can form full size aggregates once permissive conditions are present (lightning bolt) but not in areas without permissive conditions (above and below the aggregates).

In recent years, there has been a reawakening of interest into the mechanism of spreading of neurodegenerative disease from one region to another. This concept was initially proposed following a systematic study of brains of humans who died after having various clinical stages of AD (Braak and Braak, 1991). Many investigators currently hypothesize that spreading involves the movement of misfolded protein conformers, possibly as lower-order oligomers or small bits of mature aggregates (Fig. 4A, ‘Moving aggregates’). Important events that led to the recent revitalized interest in neurodegenerative disease spreading were the observation of PD neuropathology in human transplanted tissue lying adjacent to endogenous, diseased tissue (Li et al., 2008) and the experimental transmission of AD-related aggregates in mouse models (Meyer-Luehmann et al., 2006; Meyer-Luehmann et al., 2003). Combined with reports of the spreading of additional neurodegenerative-disease-related proteins (Bolmont et al., 2007; Grad et al., 2011; Harris et al., 2010; Kane et al., 2000; Luk et al., 2012), these findings have resulted in an exponential growth of interest in this topic and have fuelled discussions on whether proteins other than PrP can spread via a prion-like mechanism (Brundin et al., 2010; Cushman et al., 2010; Frost and Diamond, 2010; Guest et al., 2011; Jucker and Walker, 2011). In light of the growing number of ‘infectious’ proteins identified, the term ‘prionoid’ was coined to distinguish protein aggregates that are likely to not be naturally transmissible between individuals from those that are (prions) (Aguzzi and Rajendran, 2009). Proposed mechanisms for the spread of protein aggregates include cellular release and free diffusion through the extracellular space, transport via secreted vesicles known as exosomes (Danzer et al., 2012; Emmanouilidou et al., 2010; Lee et al., 2012; Saman et al., 2012) or movement through intercellular tunnels known as nano-tubes (Costanzo et al., 2013; Marzo et al., 2012). Mammalian prions, considered to be ‘super spreaders’ because of their unique ability to spread between individuals, have been posited to transit the brain via each of the above mechanisms (Alais et al., 2008; Gousset et al., 2009; Gousset and Zurzolo, 2009; Porto-Carreiro et al., 2005; Vella et al., 2007). It has recently been shown that cytosolic proteins can, in addition to extracellular proteins such as PrP, spread to neighboring cells via a prion-like mechanism (Hofmann et al., 2013).

A caveat of the hypothesis of disease spread being caused by the movement of aggregates is that the role of aggregates in disease remains quite contentious. It seems that some aggregates are toxic, some are benign and some might even be helpful (Kayed et al., 2003; Piccardo et al., 2007; Resenberger et al., 2011). Another problem is that, if aggregates are toxic, why is this the case? Is the mere presence of a mature aggregate toxic, or is toxicity a byproduct of the process of generating the aggregate? Do identical aggregates cause different problems in different brain regions? These are all outstanding questions that need to be resolved. Nonetheless, the presence of aggregates indicates that a non-physiological process is active in that area and a discussion of neurodegenerative disease spreading, and the corresponding aggregate pathology, is warranted even without a complete understanding of the mechanism(s) of toxicity.

Despite being well poised to flow through the brain, PrDs display somewhat restricted spreading rather than affecting the entire brain to give rise to what would be classified as a single disease. Instead, specific patterns of neural targeting are maintained. The spreading potential is partially controlled by the incorporated mutation, shown by the finding that some mutants are experimentally transmissible [for example, D178N and E200K (Tateishi et al., 1995; Tateishi and Kitamoto, 1995)], whereas others seem not to be [for example, A117V and F198S (Bugiani et al., 2000; Tateishi et al., 1996)]. Intracellular factors might also restrict spreading, such as restriction of PrP synthesis, interactions of PrP with intracellular molecules, modifications to PrP such as glycosylation or cleavage (Goh et al., 2007; Haigh et al., 2009; Kuczius and Kelsch, 2013; Moleres and Velayos, 2007), reuptake of misfolded PrP from the cell surface, or restricted production of exosomes or nano-tubes. Extracellular factors that might restrict spreading include physical barriers created by the dense mass of various cell types or perineuronal nets, extracellular chaperones or proteases, or engulfment by native phagocytic cells (micro- or astroglia). A crucial element required for significant spreading might be a breakdown of the barriers that restrict it. Therefore, if protein aggregates do indeed move through the brain, there are many variables that could influence the route and thereby the final pathological changes that accompany this movement.

An alternative explanation is that disease is caused by neurons that are adversely affected by receiving improper signaling from an earlier affected and misfiring neuron in its network (Sperling et al., 2009; Verret et al., 2012). The sum result would be the spreading of pathology. This mode of spreading is distinct from that described above in that a misfolded protein conformer, and its associated toxicity, is not physically moving. Importantly, however, in this scenario aggregates would still appear to have moved because the distribution of neurons succumbing to the stressful effects of dysfunctioning network members would expand, eventually causing degeneration and the production of aggregates (Fig. 4B, ‘Serial dysfunction’). In the case of familial neurodegenerative disease (and PrD) the mutant protein would be expressed nearly everywhere, making local *de novo* production of aggregates even more feasible because it is prone to misfolding. A fusion of these disparate hypotheses is that misfolded protein oligomers induce the conversion of neighboring proteins to misfold and form more oligomers, causing a chain reaction resembling the way a line of standing dominoes appears to move as they fall. The oligomers themselves are difficult to detect and highly transient, but they would seed the formation of aggregates and thereby cause the appearance of the spreading of aggregates. The spread would be most successful in stressed regions that are not able to degrade the oligomers (Fig. 4C, ‘Falling dominoes’). These three models have subtle but important distinctions. The ‘moving aggregate’ model posits that aggregates form in a diseased area and later move large distances into unaffected areas, inducing degenerative processes upon arrival. In the ‘serial dysfunction’ model, the aggregates never physically move but form *de novo* once an area becomes susceptible. The ‘falling dominoes’ model also requires that an area is first primed by network-dysfunction-induced stress, then oligomers act as scaffolds to seed the formation of neighboring aggregates; nonetheless, the proteins incorporated into the aggregates remain essentially in their original location. The previously cited PD-related aggregates observed in transplanted tissue (Li et al., 2008) could have developed through any of these mechanisms. Of course, additional models can be easily imagined. All of these models bring to light the following questions: how do neuronal and non-neuronal (glial, vascular, etc.) cells limit or facilitate spread? And do different misfolded proteins spread via a common mechanism or does each have its own unique strategy? PrP is emblematic of this latter question because different mutations induce different structures of aggregates in addition to targeting different regions. Therapeutic strategies designed to interfere with the toxic events related to protein misfolding will likely benefit from an understanding of how pathology spreads.

## Conclusions and future outlook

Understanding how mutant proteins target specific brain regions is clearly not a simple issue. Ultimately, the mutations carry the disease-encoding information that determines the protein conformation, but it is the interaction of mutant proteins with the combination of cell-type-specific microenvironments and brain-region-specific neural features that ultimately determines selective vulnerability. The spreading of pathology is likely controlled by a combination of opposing factors that facilitate or restrict spread. The *Prnp* knock-in lines modeling FFI and CJD mentioned above (Jackson et al., 2009; Jackson et al., 2013) will likely be a valuable tool for this issue. Each line develops pathologies in specific regions that spread into neighboring regions but not into the region primarily targeted by the opposite mutation. The separation of pathologies in the two mouse models is quite remarkable given PrP’s inherent ability to spread, and further emphasizes the point that the spreading phenomenon is likely to be more complicated than currently envisioned. An understanding of the factors that impede or facilitate spreading will make important contributions to the development of therapeutics, particularly those targeting extracellular molecules that might be more easily engaged than intracellular molecules.

These rare diseases provide a plethora of clues about the tools our brains employ to limit degenerative processes. It is up to us to decipher their meanings. As the fellow with the original Curious Case – Benjamin Button – noted, “Our lives are defined by opportunities, even the ones we miss”. Is the secret to selective vulnerability and a cure for neurodegenerative diseases right in front of us?
